# 1-(2-Ammonio­eth­yl)piperazin-1,4-diium dihydrogeno­phosphate monohydrogeno­phosphate

**DOI:** 10.1107/S160053681204189X

**Published:** 2012-10-13

**Authors:** Mohamed Lahbib Mrad, Valeria Ferretti, Mohamed Rzaigui, Cherif Ben Nasr

**Affiliations:** a Laboratoire de Chimie des Matériaux, Faculté des Sciences de Bizerte, 7021 Zarzouna, Tunisie; b Chemistry Department and Centro di Strutturistica Diffrattometrica, University of Ferrara, Via L Borsari 46, I-44121 Ferrara, Italy.

## Abstract

The structure of the title compound, C_6_H_18_N_3_·HPO_4_·H_2_PO_4_, is characterized by two kinds of inorganic chains running along the *a*-axis direction. The first one is composed of HPO_4_^2−^ anions, while the second one is built up by H_2_PO_4_^−^ anions. Both types of chains are held together by O—H⋯O hydrogen bonds. The organic cations are attached to these chains through N—H⋯O and C—H⋯O hydrogen bonds. The piperazin-1,4-diium ring adopts a chair conformation.

## Related literature

For graph-set motifs, see: Bernstein *et al.* (1995 ▶). For reference structural data, see: Kaabi *et al.* (2004 ▶); Chtioui & Jouini (2006 ▶); Jensen *et al.* (2007 ▶).
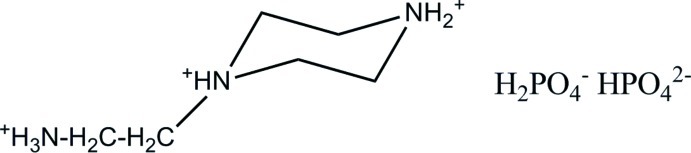


## Experimental

### 

#### Crystal data

C_6_H_18_N_3_·HPO_4_·H_2_PO_4_
*M* *_r_* = 325.20
Monoclinic, 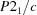

*a* = 12.9417 (2) Å
*b* = 11.1054 (2) Å
*c* = 9.3981 (4) Å
β = 92.566 (1)°
*V* = 1349.37 (7) Å^3^
*Z* = 4
Mo *K*α radiation
μ = 0.36 mm^−1^
*T* = 295 K
0.52 × 0.49 × 0.20 mm

#### Data collection

Nonius KappaCCD diffractometer
6163 measured reflections
3518 independent reflections
3167 reflections with *I* > 2σ(*I*)
*R*_int_ = 0.016

#### Refinement

*R*[*F* ^2^ > 2σ(*F* ^2^)] = 0.038
*wR*(*F* ^2^) = 0.101
*S* = 1.07
3518 reflections
256 parameters
All H-atom parameters refined
Δρ_max_ = 0.87 e Å^−3^
Δρ_min_ = −0.69 e Å^−3^

### 

Data collection: *KappaCCD Server Software* (Nonius, 1997 ▶); cell refinement: *DENZO-SMN* (Otwinowski & Minor, 1997 ▶); data reduction: *DENZO-SMN*; program(s) used to solve structure: *SIR97* (Altomare *et al.*, 1999 ▶); program(s) used to refine structure: *SHELXL97* (Sheldrick, 2008 ▶); molecular graphics: *ORTEPIII* (Burnett & Johnson, 1996 ▶); software used to prepare material for publication: *SHELXL97* and *WinGX* (Farrugia, 1999 ▶).

## Supplementary Material

Crystal structure: contains datablock(s) global, I. DOI: 10.1107/S160053681204189X/ru2043sup1.cif

Structure factors: contains datablock(s) I. DOI: 10.1107/S160053681204189X/ru2043Isup2.hkl

Additional supplementary materials:  crystallographic information; 3D view; checkCIF report

## Figures and Tables

**Table 1:** Hydrogen-bond geometry (Å, °)

*D*—H⋯*A*	*D*—H	H⋯*A*	*D*⋯*A*	*D*—H⋯*A*
N1—H1*N*⋯O3	0.93 (2)	1.66 (2)	2.582 (2)	168 (2)
N2—H2na⋯O6	0.91 (2)	1.80 (2)	2.692 (2)	167 (2)
N3—H3na⋯O3	0.87 (3)	1.90 (3)	2.759 (2)	168 (3)
O4—H4o⋯O2^i^	0.81 (4)	1.78 (4)	2.582 (2)	170 (3)
N2—H2*NB*⋯O1^i^	0.93 (3)	1.72 (3)	2.656 (2)	175 (3)
N3—H3*NB*⋯O2^ii^	0.92 (3)	1.79 (3)	2.683 (2)	164 (2)
N3—H3*NC*⋯O1^iii^	0.93 (3)	1.83 (3)	2.747 (2)	171 (2)
O8—H8*O*⋯O6^iv^	0.82 (4)	1.80 (4)	2.614 (2)	173 (4)
O7—H7*O*⋯O5^v^	0.80 (4)	1.74 (4)	2.502 (2)	159 (4)
C3—H3*A*⋯O1	0.96 (2)	2.58 (2)	3.427 (2)	147 (2)
C5—H5*B*⋯O5^vi^	0.99 (2)	2.44 (2)	3.236 (2)	137 (2)
C2—H2*B*⋯O4^vii^	0.96 (3)	2.45 (3)	3.345 (2)	154 (2)

Symmetry codes: (i) 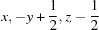; (ii) 
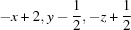
; (iii) 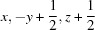; (iv) 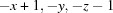; (v) 
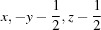
; (vi) 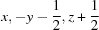; (vii) 
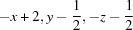
.

**Fig. 1.**
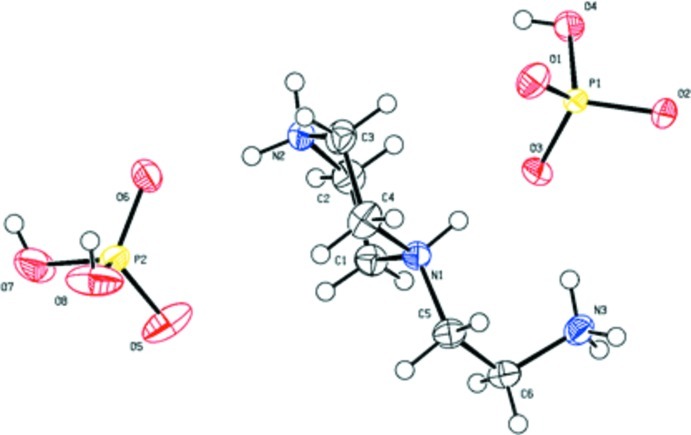
A view of the title compound, showing 50% probability displacement ellipsoids and arbitrary spheres for the H atoms.

**Fig. 2.**
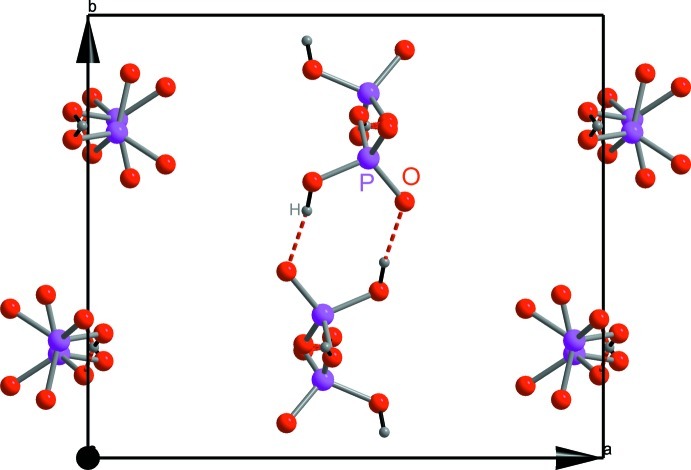
Projection along the c-axis of the inorganic chains in the structure of the title compound. PO4 is given in the tetrahedral representation. Hydrogen bonds are shown as broken lines.

**Fig. 3.**
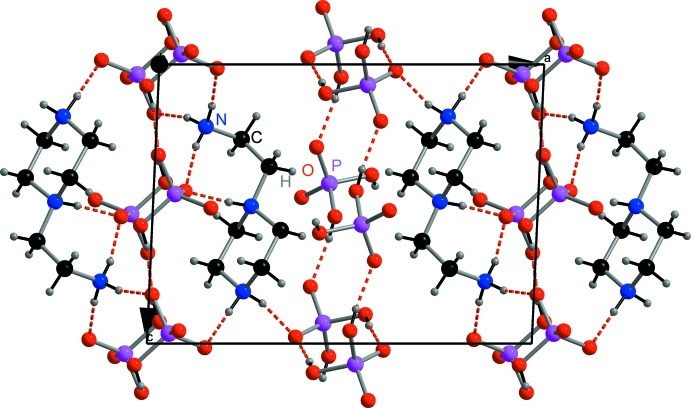
The packing diagram of the compound viewed down the b-axis. PO4 is given in the tetrahedral representation. Hydrogen bonds are shown as broken lines.

## supplementary crystallographic information

### Comment

During the systematic investigation of interaction between monophosphoric acid with organic molecules, numerous structures of monophosphates with organic cations have been described. Hydrogen bonds take part to the stability and the cohesion of the corresponding compounds. We report in this work the chemical preparation and the structural investigation of a new hydrogenomonophosphate C_6_H_18_N_3_HO_4_PH_2_O_4_P. The main feature of the atomic arrangement is the existence of two kinds of infinite chains, located at *y* = 1/4 and *y* = 3/4, and crossing the unit cell parallel to the *a*-direction. The first one is composed of HPO_4_^2-^ groups. The second one is formed by H_2_PO_4_^-^ anions and characterized by the association between the neighboring chains *via* strong O—H···O hydrogen bonds with an *R*_2_^2^(8) graph set motif (Bernstein *et al.*, 1995) centered at (1/2, 1/2, 0) (Fig. 2). The ammonioethylpiperazinediium cations are anchored onto successive chains through N—H···O and C—H···O hydrogen bonds (Fig. 3). The piperazinediium ring adopts a chair conformation and all the measured main features are similar to intramolecular bond distances and angles usually reported for such ring in hydrogenmonophosphate of organic cations (Jensen *et al.*, 2007). With regards to the geometrical features of the monophosphate anions, we remark the existence of two types of P—O distances. The shorter ones, varying between 1.488 (1) and 1.522 (1) Å, correspond to the oxygen atoms double bonded to the phosphorous atom, while the largest ones, varying between 1.556 (2) and 1.586 (1) Å, are associated with the P—OH single bond. This is in agreement with the literature data for monohydrogenophosphate anion in similar arrangements (*e.g.* Chtioui & Jouini, 2006; Kaabi *et al.*, 2004).

### Experimental

Crystals of the title compound were prepared at room temperature by slow addition of a solution of orthophosphoric acid (4 mmol in 30 ml of water) to an alcoholic solution of *N*-aminoethylpiperazine (2 mmol in 30 ml of ethanol). The acid was added until the alcoholic solution becomes turbid. After filtration, the solution was allowed to slowly evaporate at room temperature over several days leading to formation of transparent prismatic crystals with suitable dimensions for single-crystal structural analysis (yield 50%). The crystals are stable for months under normal conditions of temperature and humidity.

### Refinement

The structure was refined using full-matrix least squares with anisotropic non-H atoms. All hydrogen atoms were located in the Difference Fourier map and refined isotropically.

### Crystal data

**Table:**

C_6_H_18_N_3_·HPO_4_·H_2_PO_4_	*F*(000) = 688
*M**_r_* = 325.20	*D*_x_ = 1.601 Mg m^−^^3^
Monoclinic, *P*2_1_/*c*	Mo *K*α radiation, λ = 0.71073 Å
*a* = 12.9417 (2) Å	Cell parameters from 6163 reflections
*b* = 11.1054 (2) Å	θ = 2.0–29.0°
*c* = 9.3981 (4) Å	µ = 0.36 mm^−^^1^
β = 92.566 (1)°	*T* = 295 K
*V* = 1349.37 (7) Å^3^	Plate, pale yellow
*Z* = 4	0.52 × 0.49 × 0.20 mm

### Data collection

**Table:**

Nonius KappaCCD diffractometer	3167 reflections with *I* > 2σ(*I*)
Radiation source: fine-focus sealed tube	*R*_int_ = 0.016
Graphite monochromator	θ_max_ = 29.0°, θ_min_ = 5.1°
φ scans and ω scans	*h* = −17→17
6163 measured reflections	*k* = −15→12
3518 independent reflections	*l* = −12→12

### Refinement

**Table:**

Refinement on *F*^2^	Primary atom site location: structure-invariant direct methods
Least-squares matrix: full	Secondary atom site location: difference Fourier map
*R*[*F*^2^ > 2σ(*F*^2^)] = 0.038	Hydrogen site location: difference Fourier map
*wR*(*F*^2^) = 0.101	All H-atom parameters refined
*S* = 1.07	*w* = 1/[σ^2^(*F*_o_^2^) + (0.0441*P*)^2^ + 0.9354*P*] where *P* = (*F*_o_^2^ + 2*F*_c_^2^)/3
3518 reflections	(Δ/σ)_max_ = 0.001
256 parameters	Δρ_max_ = 0.87 e Å^−^^3^
0 restraints	Δρ_min_ = −0.69 e Å^−^^3^

### Special details

**Table:**

Geometry. All e.s.d.'s (except the e.s.d. in the dihedral angle between two l.s. planes) are estimated using the full covariance matrix. The cell e.s.d.'s are taken into account individually in the estimation of e.s.d.'s in distances, angles and torsion angles; correlations between e.s.d.'s in cell parameters are only used when they are defined by crystal symmetry. An approximate (isotropic) treatment of cell e.s.d.'s is used for estimating e.s.d.'s involving l.s. planes.
Refinement. Refinement of *F*^2^ against ALL reflections. The weighted *R*-factor *wR* and goodness of fit *S* are based on *F*^2^, conventional *R*-factors *R* are based on *F*, with *F* set to zero for negative *F*^2^. The threshold expression of *F*^2^ > σ(*F*^2^) is used only for calculating *R*-factors(gt) *etc*. and is not relevant to the choice of reflections for refinement. *R*-factors based on *F*^2^ are statistically about twice as large as those based on *F*, and *R*- factors based on ALL data will be even larger.

### Fractional atomic coordinates and isotropic or equivalent isotropic displacement parameters (Å^2^)

**Table:**

	*x*	*y*	*z*	*U*_iso_*/*U*_eq_	
P1	0.94393 (3)	0.26513 (3)	0.03610 (4)	0.01932 (11)	
P2	0.54421 (3)	−0.17696 (4)	−0.42663 (4)	0.02605 (12)	
N1	0.74513 (10)	0.02204 (12)	−0.00827 (14)	0.0225 (3)	
N2	0.75588 (12)	0.04193 (13)	−0.31310 (15)	0.0284 (3)	
N3	0.87175 (12)	−0.00772 (13)	0.27737 (16)	0.0275 (3)	
O1	0.84923 (10)	0.33714 (11)	−0.01467 (13)	0.0314 (3)	
O2	0.99197 (11)	0.31318 (11)	0.17482 (12)	0.0322 (3)	
O3	0.91834 (10)	0.13172 (10)	0.04589 (13)	0.0298 (3)	
O4	1.02998 (10)	0.28122 (12)	−0.07732 (14)	0.0319 (3)	
O5	0.58176 (14)	−0.24304 (15)	−0.29656 (16)	0.0517 (4)	
O6	0.61467 (9)	−0.07876 (11)	−0.47656 (13)	0.0304 (3)	
O7	0.52603 (19)	−0.27294 (18)	−0.54576 (19)	0.0687 (7)	
O8	0.43568 (12)	−0.12345 (16)	−0.3960 (2)	0.0548 (5)	
C1	0.78068 (14)	−0.08225 (14)	−0.09447 (19)	0.0280 (3)	
C2	0.82930 (14)	−0.03456 (16)	−0.22692 (19)	0.0306 (3)	
C3	0.71060 (14)	0.13880 (16)	−0.22738 (19)	0.0313 (3)	
C4	0.66558 (14)	0.09023 (18)	−0.09418 (19)	0.0323 (4)	
C5	0.70292 (13)	−0.01058 (16)	0.13353 (18)	0.0287 (3)	
C6	0.77789 (14)	−0.07807 (16)	0.23154 (19)	0.0305 (3)	
H1A	0.833 (2)	−0.124 (2)	−0.042 (3)	0.047 (7)*	
H2NA	0.7042 (19)	−0.002 (2)	−0.356 (3)	0.040 (6)*	
H4O	1.011 (3)	0.250 (3)	−0.152 (4)	0.068 (9)*	
H1B	0.7218 (18)	−0.130 (2)	−0.118 (2)	0.035 (6)*	
H3NA	0.895 (2)	0.033 (2)	0.207 (3)	0.050 (7)*	
H3NB	0.921 (2)	−0.062 (2)	0.309 (3)	0.045 (7)*	
H3NC	0.8586 (19)	0.045 (2)	0.351 (3)	0.043 (6)*	
H2NB	0.790 (2)	0.080 (2)	−0.386 (3)	0.052 (7)*	
H2A	0.8872 (18)	0.015 (2)	−0.204 (2)	0.036 (6)*	
H2B	0.8482 (19)	−0.099 (2)	−0.288 (3)	0.048 (7)*	
H3A	0.7650 (19)	0.195 (2)	−0.203 (3)	0.041 (6)*	
H3B	0.6571 (18)	0.177 (2)	−0.284 (3)	0.037 (6)*	
H4A	0.6092 (19)	0.037 (2)	−0.114 (3)	0.040 (6)*	
H5A	0.6835 (18)	0.066 (2)	0.181 (3)	0.039 (6)*	
H6A	0.7415 (19)	−0.098 (2)	0.314 (3)	0.043 (6)*	
H6B	0.803 (2)	−0.153 (2)	0.184 (3)	0.048 (7)*	
H5B	0.6421 (16)	−0.0630 (19)	0.117 (2)	0.030 (5)*	
H4B	0.6433 (19)	0.154 (2)	−0.038 (3)	0.045 (7)*	
H8O	0.425 (3)	−0.058 (3)	−0.435 (4)	0.082 (11)*	
H7O	0.539 (3)	−0.250 (3)	−0.623 (5)	0.089 (12)*	
H1N	0.8034 (18)	0.071 (2)	0.006 (2)	0.038 (6)*	

### Atomic displacement parameters (Å^2^)

**Table:**

	*U*^11^	*U*^22^	*U*^33^	*U*^12^	*U*^13^	*U*^23^
P1	0.02330 (19)	0.01977 (18)	0.01494 (17)	−0.00404 (13)	0.00127 (13)	−0.00001 (12)
P2	0.0326 (2)	0.0265 (2)	0.0191 (2)	−0.00124 (15)	0.00252 (15)	0.00181 (14)
N1	0.0219 (6)	0.0237 (6)	0.0220 (6)	−0.0004 (5)	0.0016 (5)	−0.0002 (5)
N2	0.0319 (7)	0.0325 (7)	0.0209 (6)	−0.0090 (6)	0.0004 (5)	−0.0021 (5)
N3	0.0332 (7)	0.0263 (6)	0.0226 (6)	0.0012 (5)	−0.0019 (5)	0.0025 (5)
O1	0.0348 (6)	0.0338 (6)	0.0255 (6)	0.0089 (5)	0.0004 (5)	−0.0002 (5)
O2	0.0455 (7)	0.0336 (6)	0.0170 (5)	−0.0129 (5)	−0.0029 (5)	−0.0019 (4)
O3	0.0348 (6)	0.0224 (5)	0.0316 (6)	−0.0089 (4)	−0.0036 (5)	0.0040 (4)
O4	0.0289 (6)	0.0444 (7)	0.0228 (6)	−0.0119 (5)	0.0065 (5)	−0.0030 (5)
O5	0.0701 (10)	0.0554 (9)	0.0303 (7)	0.0276 (8)	0.0105 (7)	0.0157 (6)
O6	0.0274 (6)	0.0297 (6)	0.0339 (6)	−0.0049 (5)	−0.0014 (5)	−0.0016 (5)
O7	0.1099 (16)	0.0634 (11)	0.0350 (8)	−0.0542 (11)	0.0256 (9)	−0.0179 (8)
O8	0.0350 (7)	0.0520 (9)	0.0790 (12)	0.0093 (7)	0.0197 (7)	0.0335 (9)
C1	0.0311 (8)	0.0220 (7)	0.0310 (8)	0.0022 (6)	0.0035 (6)	−0.0020 (6)
C2	0.0292 (8)	0.0323 (8)	0.0309 (8)	0.0018 (6)	0.0067 (6)	−0.0057 (7)
C3	0.0359 (9)	0.0280 (8)	0.0293 (8)	0.0033 (7)	−0.0058 (7)	0.0012 (6)
C4	0.0275 (8)	0.0388 (9)	0.0306 (8)	0.0105 (7)	0.0010 (6)	−0.0004 (7)
C5	0.0277 (7)	0.0330 (8)	0.0259 (8)	−0.0015 (6)	0.0066 (6)	0.0005 (6)
C6	0.0372 (9)	0.0290 (8)	0.0255 (8)	−0.0050 (7)	0.0027 (6)	0.0048 (6)

### Geometric parameters (Å, º)

**Table:**

P1—O2	1.5160 (12)	N3—H3NC	0.93 (3)
P1—O3	1.5218 (11)	O4—H4O	0.81 (3)
P1—O1	1.5222 (12)	O7—H7O	0.80 (4)
P1—O4	1.5858 (12)	O8—H8O	0.82 (4)
P2—O5	1.4886 (14)	C1—C2	1.515 (2)
P2—O6	1.5097 (12)	C1—H1A	0.94 (3)
P2—O8	1.5635 (16)	C1—H1B	0.95 (2)
P2—O7	1.5561 (17)	C2—H2A	0.95 (2)
N1—C4	1.487 (2)	C2—H2B	0.96 (3)
N1—C1	1.498 (2)	C3—C4	1.504 (3)
N1—C5	1.507 (2)	C3—H3A	0.96 (3)
N1—H1N	0.93 (2)	C3—H3B	0.95 (2)
N2—C3	1.481 (2)	C4—H4A	0.96 (2)
N2—C2	1.487 (2)	C4—H4B	0.94 (3)
N2—H2NA	0.91 (3)	C5—C6	1.507 (2)
N2—H2NB	0.93 (3)	C5—H5A	0.99 (2)
N3—C6	1.492 (2)	C5—H5B	0.99 (2)
N3—H3NA	0.87 (3)	C6—H6A	0.95 (3)
N3—H3NB	0.92 (3)	C6—H6B	1.01 (3)
			
O2—P1—O3	111.88 (7)	C2—C1—H1A	107.2 (16)
O2—P1—O1	112.21 (7)	N1—C1—H1B	107.5 (14)
O3—P1—O1	110.86 (7)	C2—C1—H1B	111.2 (14)
O2—P1—O4	105.37 (7)	H1A—C1—H1B	113 (2)
O3—P1—O4	108.16 (7)	N2—C2—C1	111.62 (14)
O1—P1—O4	108.06 (7)	N2—C2—H2A	105.7 (14)
O5—P2—O6	115.58 (10)	C1—C2—H2A	111.8 (14)
O5—P2—O8	107.41 (9)	N2—C2—H2B	105.8 (15)
O6—P2—O8	110.04 (8)	C1—C2—H2B	111.1 (16)
O5—P2—O7	106.64 (12)	H2A—C2—H2B	111 (2)
O6—P2—O7	110.19 (8)	N2—C3—C4	111.70 (14)
O8—P2—O7	106.54 (13)	N2—C3—H3A	107.2 (15)
C4—N1—C1	108.79 (13)	C4—C3—H3A	110.2 (15)
C4—N1—C5	109.46 (13)	N2—C3—H3B	108.0 (14)
C1—N1—C5	115.14 (12)	C4—C3—H3B	109.1 (15)
C4—N1—H1N	108.7 (14)	H3A—C3—H3B	111 (2)
C1—N1—H1N	105.1 (14)	N1—C4—C3	110.49 (14)
C5—N1—H1N	109.5 (15)	N1—C4—H4A	107.1 (14)
C3—N2—C2	112.18 (13)	C3—C4—H4A	112.3 (15)
C3—N2—H2NA	109.3 (15)	N1—C4—H4B	107.4 (16)
C2—N2—H2NA	111.8 (15)	C3—C4—H4B	109.9 (16)
C3—N2—H2NB	106.3 (17)	H4A—C4—H4B	109 (2)
C2—N2—H2NB	110.2 (16)	N1—C5—C6	114.28 (14)
H2NA—N2—H2NB	107 (2)	N1—C5—H5A	107.5 (14)
C6—N3—H3NA	111.2 (17)	C6—C5—H5A	108.6 (14)
C6—N3—H3NB	107.3 (16)	N1—C5—H5B	108.5 (13)
H3NA—N3—H3NB	109 (2)	C6—C5—H5B	107.0 (12)
C6—N3—H3NC	111.9 (15)	H5A—C5—H5B	111.0 (19)
H3NA—N3—H3NC	109 (2)	N3—C6—C5	114.18 (14)
H3NB—N3—H3NC	109 (2)	N3—C6—H6A	108.0 (15)
P1—O4—H4O	110 (2)	C5—C6—H6A	106.6 (15)
P2—O7—H7O	114 (3)	N3—C6—H6B	106.4 (15)
P2—O8—H8O	113 (3)	C5—C6—H6B	110.8 (15)
N1—C1—C2	108.85 (13)	H6A—C6—H6B	111 (2)
N1—C1—H1A	109.1 (16)		

### Hydrogen-bond geometry (Å, º)

**Table:**

*D*—H···*A*	*D*—H	H···*A*	*D*···*A*	*D*—H···*A*
N1—H1*N*···O3	0.93 (2)	1.66 (2)	2.582 (2)	168 (2)
N2—H2na···O6	0.91 (2)	1.80 (2)	2.692 (2)	167 (2)
N3—H3na···O3	0.87 (3)	1.90 (3)	2.759 (2)	168 (3)
O4—H4o···O2^i^	0.81 (4)	1.78 (4)	2.582 (2)	170 (3)
N2—H2*NB*···O1^i^	0.93 (3)	1.72 (3)	2.656 (2)	175 (3)
N3—H3*NB*···O2^ii^	0.92 (3)	1.79 (3)	2.683 (2)	164 (2)
N3—H3*NC*···O1^iii^	0.93 (3)	1.83 (3)	2.747 (2)	171 (2)
O8—H8*O*···O6^iv^	0.82 (4)	1.80 (4)	2.614 (2)	173 (4)
O7—H7*O*···O5^v^	0.80 (4)	1.74 (4)	2.502 (2)	159 (4)
C3—H3*A*···O1	0.96 (2)	2.58 (2)	3.427 (2)	147 (2)
C5—H5*B*···O5^vi^	0.99 (2)	2.44 (2)	3.236 (2)	137 (2)
C2—H2*B*···O4^vii^	0.96 (3)	2.45 (3)	3.345 (2)	154 (2)

Symmetry codes: (i) *x*, −*y*+1/2, *z*−1/2; (ii) −*x*+2, *y*−1/2, −*z*+1/2; (iii) *x*, −*y*+1/2, *z*+1/2; (iv) −*x*+1, −*y*, −*z*−1; (v) *x*, −*y*−1/2, *z*−1/2; (vi) *x*, −*y*−1/2, *z*+1/2; (vii) −*x*+2, *y*−1/2, −*z*−1/2.
